# Bond Behavior of a Bio-Aggregate Embedded in Cement-Based Matrix

**DOI:** 10.3390/ma15176151

**Published:** 2022-09-05

**Authors:** Saulo Rocha Ferreira, Rodolfo Giacomim Mendes de Andrade, Gabriele Melo de Andrade, Olga Maria Oliveira de Araújo, Ricardo Tadeu Lopes, Eduardo de Moraes Rego Fairbairn, Thiago Melo Grabois, Neven Ukrainczyk

**Affiliations:** 1 Civil Engineering Department, Federal University of Lavras, C.P.3037, Lavras 37200-900, MG, Brazil; 2 Civil Engineering and Buildings Department, Instituto Federal do Espírito Santo, Av. Vitória, 1729, Vitória 29040-780, ES, Brazil; 3 Nuclear Engineering Department, COPPE, Universidade Federal do Rio de Janeiro, P.O. Box 68506, Rio de Janeiro 21941-972, RJ, Brazil; 4 Universidade Federal do Rio de Janeiro, Avenida Pedro Calmon, 550, Rio de Janeiro 21941-901, RJ, Brazil; 5 Institute of Construction and Building Materials, TU Darmstadt, Franziska-Braun-Strasse 3, 64287 Darmstadt, Germany

**Keywords:** macaúba endocarp, bio-aggregate, lightweight concrete, mechanical properties, image analysis, numerical modeling

## Abstract

This paper investigates the bond behavior between a bio-aggregate and a cement-based matrix. The experimental evaluation comprised physical, chemical, image, and mechanical characterization of the bio-aggregate. The image analyses about the bio-aggregate’s outer structure provided first insights to understand the particularities of this newly proposed bio-aggregate for use in cementitious materials. A mineral aggregate (granitic rock), largely used as coarse aggregate in the Brazilian civil construction industry, was used as reference. The bond behavior of both aggregates was evaluated via pull-out tests. The results indicated that both aggregates presented a similar linear elastic branch up to each respective peak loads. The peak load magnitude of the mineral aggregate indicated a better chemical adhesion when compared to the bio-aggregate’s. The post-peak behavior, however, indicated a smoother softening branch for the bio-aggregate, corroborated by the microscopy image analyses. Although further investigation is required, the macaúba crushed endocarp was found to be a thriving bio-material to be used as bio-aggregate.

## 1. Introduction

The development of non-conventional materials has been allowing one to design an array of novel structural elements that can be subjected to different environmental and loading conditions. For instance, bio-based aggregates, such as coffee husk [1], bamboo, rice and wood particles [2,3], allow the production of lightweight bio-concretes [3]. Other approaches can be made to produce eco-friendly materials, such as recycled aggregates [4] and artificial geopolymer aggregates [5]. For instance, artificial aggregates allow the application of several residues, such as industrial residues of coal fly ash [6], palm oil fly ash [7], and bottom ash [8] and present good properties. In engineered cementitious composites, artificial aggregates present good matrix-aggregate interface, improving crack induction and strength [5]. Some interesting properties include the improved thermal and noise insulation capacity as well as the decrease in the structure’s dead load on foundations. The evolution of cement-based composites is related to the binder constituents optimization and hence the cementitious matrix-aggregate interface, which affects from regular to ultra-high performance cement-based materials. The increase in packing density of the interfacial transition zone (ITZ) between cement matrix and aggregate plays an import role in the development of required mechanical properties and improved durability for the material [9,10,11,12,13,14].

Although the discussion about ITZ has been covered in the past years [15,16,17,18,19,20,21,22,23,24,25,26], standard procedures—reported from the technical committees and task groups—are still not adapted to consider bio-aggregates. Of particular interest, Mindess [14] proposed a methodology to characterize the mechanical properties in the ITZ of concretes. In this context, considering the lack of standardized methods, researchers may have inconsistency when comparing their findings, since different boundary conditions can be set up while carrying out those tests [9].

**Figure 1:**
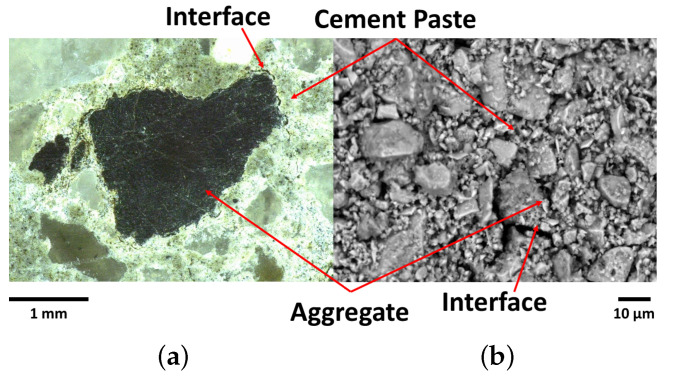
Group 1: investigation of the interactions between the aggregate and cement-based matrices by image analyzes: zommed-in views for (**a**) macaúba endocarp and (**b**) river sand embedded in cement paste.

Table 1 summarizes some of the procedures previous reported in the literature to analyze and evaluate the properties of the cement matrix-aggregate interface. It is organized in three groups. In the first one, the analyses are focused on the sub-micro and micro-structure of the matrix-aggregate ITZ. They indicated that the ITZ may be the composite deficiency mainly affected by its low chemical forces and its porosity [15]. The ITZ can be qualitatively investigated by visual tools (Figure 1), such as scanning electronic microscopy (SEM), or quantitatively by instruments, e.g., nano or micro indentations that can infer the interfacial stiffness [16].

**Figure 2:**
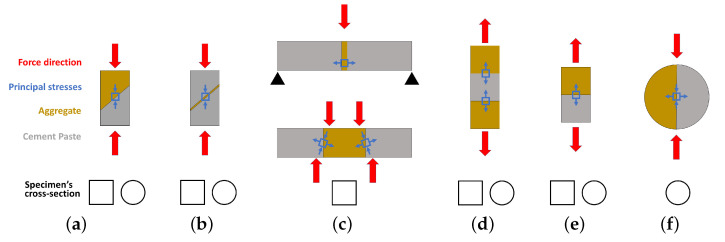
Group 2: Typical tests to investigate the mechanical behavior between aggregate and cement-based matrices: (**a**,**b**) slant shear—adapted from [17], (**c**) flexural (top) and shear (bottom)—adapted from [25]), (**d**,**e**) direct tensile, and (**f**) indirect tensile—adapted from [19]) *apud* [18]).

The research presented as Group 2 focused on the interaction mechanics between the aggregate and the cement-based matrix. These investigations dealt with the interface region subjected to different loading conditions, e.g., tensile, shear, and bending [16]. However, as depicted in Figure 2, most of the experiments required geometrical adjustments to the aggregate through polishing procedures, shattering, saw-cutting, among others, therefore modifying its natural characteristics. For instance, when polishing and saw-cutting the aggregate its roughness and morphology considerably decreases, resulting in a limited physical and chemical evaluation of the matrix-aggregate interaction [17,18,19].

**Figure 3:**
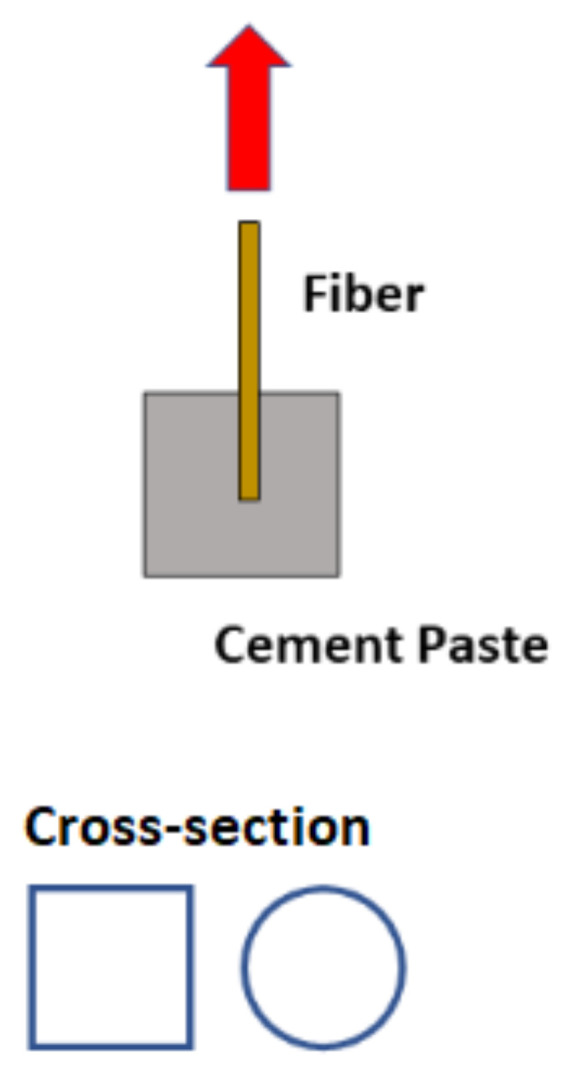
Group 3: Typical pull-out test configuration of a fiber element embedded in a cement-based matrix.

Finally, in Group 3, the studies proposed empirical analyses from experiments that are not included in the discussion provided by the references of Groups 1 and 2. Furthermore, the aggregates were employed without any modifications (see Figure 3). For instance, the axial compressive test or indirect tensile test can provide relevant information regarding aggregate-matrix bond, i.e., if the aggregate’s surface presents low bond to the cement-based matrix, the composite’s fracture mode and compressive strength will indirectly indicate so. As these tests provide bond quality only, there is still no agreement amongst researchers on how to quantify the aggregate-matrix adhesion [22].

Overall, despite the interesting aspects of many of the alternatives demonstrated to evaluate the bond between aggregate and cement matrix, the aggregate modifications in such characteristics as shape, size, and roughness presented relevant effects on the mechanical response. The lack of such experimental procedure is the main motivation of this research. Hence, performing a test with a single aggregate with its original characteristics would considerably help to understand its interaction with the cement matrix in a more reliable way. Therefore, this paper presents an adaptation of a pull-out test performed with a single fiber or aggregate.

## 2. Materials and Methods

The materials used in this work comprised bio-aggregates, Mauá/LafargeHolcim’s cement Type V-ARI (equivalent to the European CEM I 52.5N [27], from Rio de Janeiro, Brazil —its chemical composition is shown in Table 2), mineral coarse aggregates (oblong-shaped crushed granite), and distilled water.

**Figure 4:**
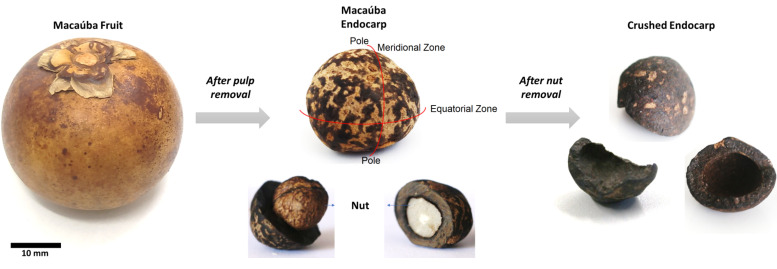
Bio-aggregate: from Macaúba fruit to crushed endocarp.

The bio-aggregate used in this research is originated from the Macaúba fruit, as shown in Figure 4. The crushed endocarp was donated by G-OLEO, a research group from the Federal University of Lavras, Brazil. Two parts of the Macaúba fruit are commercially of interest: its pulp and its nut. Thus, after pulp extraction, the endocarp is submitted to a high-speed centrifuge, which provides the required nut, and its residue, the crushed endocarp.

### 2.1. Physical-Chemical Analyses

#### 2.1.1. Particle Size Distribution

For coarse aggregates sieve analysis, the standard ASTM C136 (2014) [28] was used. The test was used to determinate the aggregates’ size distribution by sieving. Regarding cement analysis, the particle size distribution were determined using a HORIBA LA-950V2 (RETSCH GmbH, from Hann, Germany) type laser particle size analyzer. During the measurement ultrasonic treatment was used for the dispersion of fine particles in ethanol. Three tests were carried out with refraction indexes of 1, 1.35 and 1.7.

#### 2.1.2. Density and Water Absorption of the Bio-Aggregates

Firstly, the bio-aggregates were cleaned in running water, followed by a manual scrubbing to remove residual mucilage. For their basic density and water absorption capacity, an adapted version of ASTM D2395-17 [29] was considered, i.e., the bio-aggregates were submerged in water to saturation point, and had their masses and saturated volumes measured. Subsequently, for 72 h they were kept in the oven at 103 ± 2 ${}^{{}^{\circ}}$C to have their dry masses measured.

#### 2.1.3. Semi-Adiabatic Calorimetry

The calorimetry analysis was conducted in a multichannel semi-adiabatic calorimeter, build with a datalogger PIKO loger TC-08 manufactured by Pico Technology, from Cambridgeshire, UK. The heat analysis was performed on cement pastes hydrated at 25 ${}^{{}^{\circ}}$C (with a precision of 0.001 ${}^{{}^{\circ}}$C) with K-type thermos couples with 32 AWG for 20 h. A one-minute manual mixing was performed with a hand vortex (by Heidolph Instruments GmbH & Co. KG, from Schwabach, Germany) at 5600 rpm. About 500 g of cement were used for the analysis.

### 2.2. Chemical Analyses

The total extractives were quantified from an adaptation of the standard TAPPI T204 cm-17 [30]. With a Soxhlet extractor (by mylabor, from São Paulo, Brazil), acetone was removed after a five-hour procedure, followed by a final wash with water at 80 ${}^{{}^{\circ}}$C. The TAPPI T211 om-16 [31] procedure was applied to determine the ash content. Samples were heated to 525 ${}^{{}^{\circ}}$C in a muffle furnace, where they remained at this temperature for three hours. The insoluble lignin was quantified according to standard TAPPI T222 om-15 [32] by the hydrolysis method. The holocellulose content was obtained following the procedure described in a 1963 report, by Browning [33].

### 2.3. Image Analyses

#### 2.3.1. Scanning Electron Microscopy

A general view of the Endocarp microstructure was investigated using an enviromental SEM ZEISS LEO EVO LS 25 (from Oberkochen, Germany). The microscope was operated under an accelerating voltage of 15 kV with 2 nA probe current. No pre-coating was applied. Specimens were fixed in a metal stub covered by a carbon-coated tape. The analysis was performed in a high vacuum and a working distance of 8 mm. No tilt was applied. Both backscattering and secondary electrons detectors were used.

#### 2.3.2. Light and Laser Microscopy

Optical micrographs were obtained using a VHX-600 digital light microscope (by Keyence, from Osaka, Japan), with VH-z20R Keyence lens with magnification of 20–200×, while a LEXT OLS 4000 laser microscope (by Olympus IMS, from Waltham, MA, USA) was used to obtain roughness and topography profile of studied aggregates, with objective lenses with magnification of 20 and 50×.

#### 2.3.3. MicroCT Scan

For microCT scan, the system acquisition was a V-Tomex-M (GE Measurement & Control Solutions, Wunstorf, Germany). The selected parameters for each sample of this work were: voltage of 100 kV, current of 180 μA, exposure time of 300 ms per projection, 7 frames, magnification of 10.43, pixel size and voxel size of 19 μm, and a total of 1500 images. The 3D reconstructions were performed using the Phoenix Datos software (by GE, from Boston, MA, USA), in which the slice alignment, beam hardening correction and were implemented and a mathematical edge-enhancement filter was applied in order to achieve a better contrast between the rock matrix and the pores. For the 3D and 2D visualization, VG Studio Max v. 3.0 (by Volume Graphics GmbH, from Heidelberg, Germany) software was used.

### 2.4. Mechanical Analyses

#### 2.4.1. Compressive Strength Test of the Cement Paste

The mixtures were prepared in a laboratory with controlled environment, i.e., room temperature of 21 ± 1 ${}^{{}^{\circ}}$C and relative humidity (RH) of 60 ± 5%. A cement paste with water-cement ratio of 0.3 was prepared with a mixerat 2000 rpm (by Heidolph Instruments GmbH & Co. KG, from Schwabach, Germany). The mix was then cast into 40 × 40 × 40 mm${}^{3}$ molds, in accordance with BS EN 196-1 [34], but without using sand aggregates, and cured at 100% RH.

The tests were carried out at 7 days of age, using a displacement rate of 0.4 MPa/s according to BS EN 12390-3 [35]. Compressive tests of cube specimens were performed in an electromechanical testing machine developed by AROTEC Time Group Inc., made in Beijing, People’s Republic of China, with a load cell of 20 kN.

#### 2.4.2. Compressive Strength Test of the Semi Ellipsoidal-Shaped Bio-Aggregate

**Figure 5:**
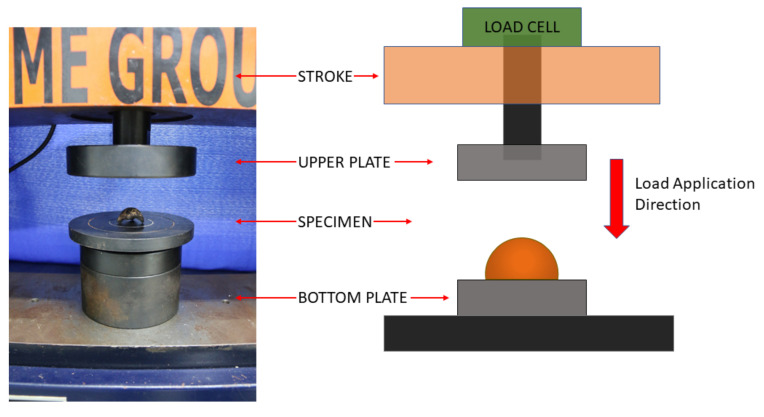
Crushed endocarp specimen during compressive strength test.

Typical semi ellipsoidal-shaped crushed endocarps (see Figure 4) were subjected to compressive tests, with each sample presenting its circular base facing the lower plate of the machine, as depicted in Figure 5. The same machinery setup described in Section 2.4.1 was used for the bio-aggregate tests, but with a displacement rate control of 0.08 mm/s instead.

#### 2.4.3. Pull-Out Test of the Aggregates

**Figure 6:**
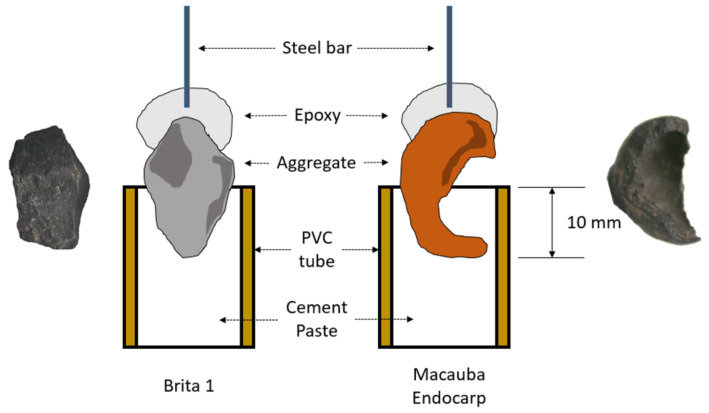
Aggregate pull-out test setup: crushed granite (**left**) and semi ellipsoidal-shaped crushed endocarp (**right**).

The bonding between aggregates (granitic rock and macaúba endocarp) and cement-based matrix was evaluated via an adapted pull-out test [1], whose setup is shown in Figure 6. With an embedment length of 10 mm, the aggregates were fixed on the machine grips by a 6.35 mm diameter round SAE 1010 steel bar epoxy (Sikadur-32, by Sika, Baar, Switzerland) glued on the top of each aggregate.

Since bond strength in pull-out tests with natural fibers does not significantly increase when comparing to 14, 21 and 28 day-old tests, the tests were carried out when the cement paste was cured for 7 days. Twelve samples were used for each test.

## 3. Numerical Modeling

To simulate the mechanical behavior of the pull-out tests, a three-dimensional nonlinear analysis was carried out using the finite element software DIANA 10.5 (DIsplacement ANAlyzer). The complex shape of the macaúba endocarp required some simplifications to be taken into account when developing this numerical model. Therefore, a semi ellipsoidal-shaped shell geometry with constant thickness was adopted for the endocarp. For that, a solid of revolution was necessaty, i.e., imported coordinates from an outer ellipse (a=23.2 mm and b=21.4 mm) and an inner ellipse (a=21.6 mm and b=19.8 mm) generated a plane image, which was rotated around an axis parallel to Z-axis to create the shell. This shell was then embedded in a cylinder with a diameter and height equal to 40.0 mm.

### 3.1. Mesh, Element Types and Materials

**Figure 7:**
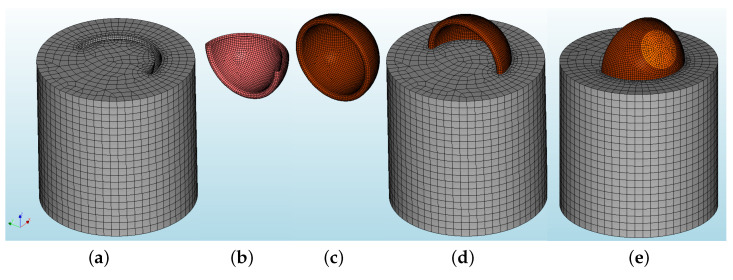
Numerical model’s mesh: (**a**) cement paste cylinder, (**b**) endocarp-cement paste interface, (**c**) crushed endocarp, (**d**) complete model (front), and (**e**) complete model (back).

The model was meshed with a seeding method based on elements’ size, which provided 4 mm-sided elements for the cylinder (Figure 7a) and 1 mm-sided elements for the bio-aggreate-cylinder interface (Figure 7b) and the bio-aggregate (Figure 7c). Figure 7d shows the complete model (front) while Figure 7e shows, in light brown, the area where the vertical loading is applied.

For the cylinder and the bio-aggregate simulation, the following elements were used [36]: *CTE30* (a ten-node, three-side isoparametric solid tetrahedron element, based on quadratic interpolation and numerical integration), *CPY39* (a thirteen-node isoparametric solid pyramid element, based on quadratic interpolation and integration), *CTP45* (a fifteen-node isoparametric solid wedge element, based on quadratic interpolation and numerical integration), and *CHX60* (a twenty-node isoparametric solid brick element, based on quadratic interpolation and Gauss integration). The zero-thickness elements for the bio-aggregate-cylinder interface were the *CT36I* (plane triangle based on quadratic interpolation, with a 6-point integration scheme), and the *CQ48I* (plane quadrilateral based on quadratic interpolation, with a 4×4 Newton-Cotes integration scheme) elements.

Since no cracks of the bio-aggregate were reported, it was regarded as a regular linear elastic material ($E_{bio}$ = 2500 MPa and $\nu_{bio}=0.2$), while the physical nonlinear properties of the cylinder’s cement paste are shown in Table 3. Amongst the material models for interface elements found in DIANA 10.5, the Coulomb Friction model was found to satisfactorily represent the shearing behavior between the bio-aggregate and the cement paste; the input parameters are presented in Table 4.

### 3.2. Boundary Conditions, Load and Nonlinear Analysis

As for the experimental setup, which had the base of the cylinder fixed on a grip, the cylinder had its bottom nodes fixed in all three translation directions, i.e., X-, Y-, and Z-axes. Moreover, on the top-back surface of the endocarp, highlighted in light brown (Figure 7e), due to the type of applied loading, vertical fixed translation supports were inserted (Z-axis). Then, a vertical imposed displacement was applied on the highlighted surface of the endocarp; a total displacement of 1.4 mm was chosen to match the magnitude of the experimental displacement.

For the nonlinear analysis, an incremental-iterative solution procedure was adopted, using displacement control for the incremental procedure (50 0.02-sized load steps were used), and Secant Method (Quasi-Newton) for the iterative procedure; the maximum number of iterations per load step was set to 25, with an energy tolerance of $10^{-4}$.

## 4. Results and Discussion

Aggregates’ surface and shape can directly interfere on the adherence with the cementitious matrix, as both physical properties affect friction and mechanical interaction with a cementitious matrix. Moreover, the aggregate’s chemical composition affects adhesion to the matrix. Thus, the following results and discussion are presented on this matter.

### 4.1. Physical-Chemical Analyses

#### 4.1.1. Dimensions of the Aggregates

**Figure 8:**
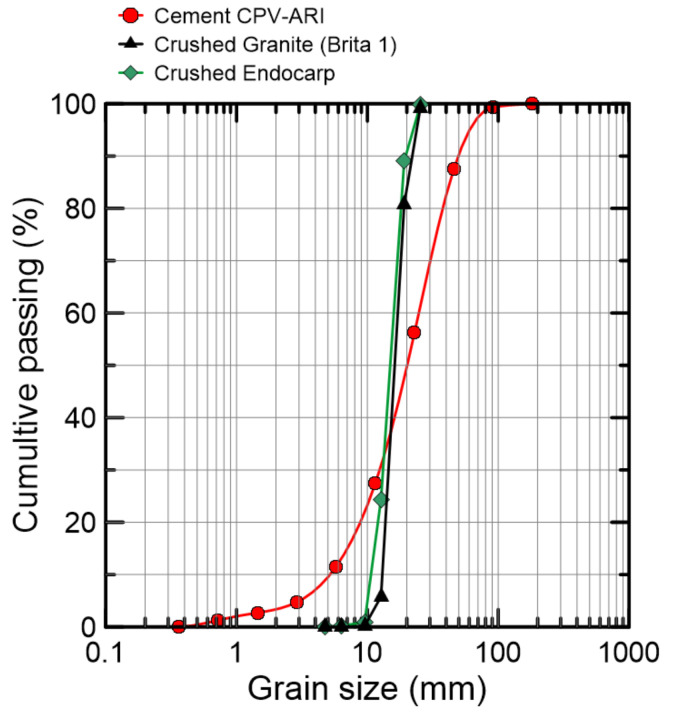
Particle size distribution of crushed granite and endocarp.

The particle size distribution of the bio-agrgegate crushed endocarp, as well as the cement’s and the mineral aggregate’s (Brazilian coarse aggregate type *Brita 1* is equivalent to Type 2 (BS EN 12620 [37]) and Type 7 (ASTM C 33 [38]).), are presented in Figure 8. The aggregates dimension analysis indicate that both aggregates present elongated shape, based on relations amongst thickness, length and width (see Table 5). Despite the granulometric variation, specimens with similar geometry were hand-picked for the pull-out test. To match the bio-aggregate’s elongated geometry, a mineral aggregate with similar geometry was selected.

#### 4.1.2. Density and Water Absorption of Crushed Endocarp

The results obtained for the basic density and the absorption of water are shown in Table 6. Following the classification of Syofyan et al. [39], the macaúba endocarp (a lignocelulosica-based material) can have its density classified as *very heavy*, since its value is greater than 1 g/cm${}^{3}$. Regarding conventional cement-based materials, the value of bulk density of aggregates commonly used in normal-weight concretes is 2.7 g/cm${}^{3}$; the specific gravity of regular aggregates ranges from 2.4 to 2.9 g/cm${}^{3}$.

Coarse and fine aggregates generally present absorption levels (moisture contents at Saturated Surface Dry—SSD) between 0.2% and 3.0%, and 0.2% and 2.0%, respectively. Lightweight aggregates present a higher absorption level, between 10 and 20%, mainly caused by the presence of pores [40,41,42]. Aggregates with high absorption levels tend to have high drying shrinkage, specially lightweight aggregates, which is approximately 0.035%, according to the BS EN 1992-1-1 [43]. From Table 6, macaúba’s drying shrinkage is 6.75%, lower than other usual bio-agregates, such as wood particles (10%) and coffee husk (23%) [44].

#### 4.1.3. Semi-Adiabatic Calorimetry

**Figure 9:**
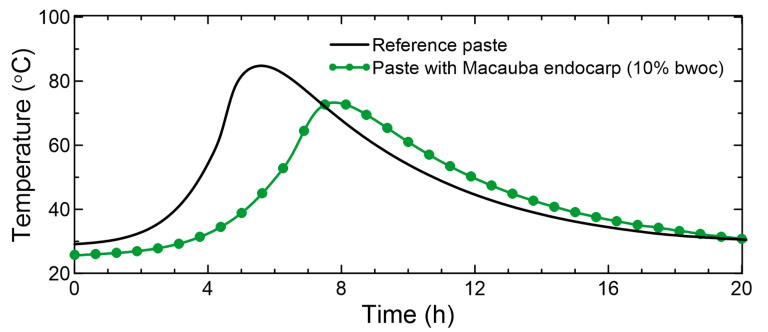
Temperature curves from the semi-adiabatic calorimetry test for reference cement paste and paste with crushed endocarp.

Figure 9 shows the temperature curves from the semi-adiabatic calorimetry test of cement pastes produced with and without macauba endocarp. The results demonstrate that the presence of the endocarp bio-aggregate delayed the hydration process of cement, with the induction period starting approximately 2 h after the reference paste.

#### 4.1.4. Chemical Analyses

The results obtained from the chemical analysis shown in Table 7 indicated a very low content of extractives (3.10%) when compared to an average content in hardwood and softwood. Since these are components of low molecular weight, this result corroborates with other indicators of high density for wood and lignocelllulosic-based materials [2]. This low content of extractives allow this material to be used in industrial scale, as it implies no need for pre-treatment of the endocarp when using in cementitious composites [3,45,46].

### 4.2. Image Analyses

#### 4.2.1. Light and Laser Microscopy

**Figure 10:**
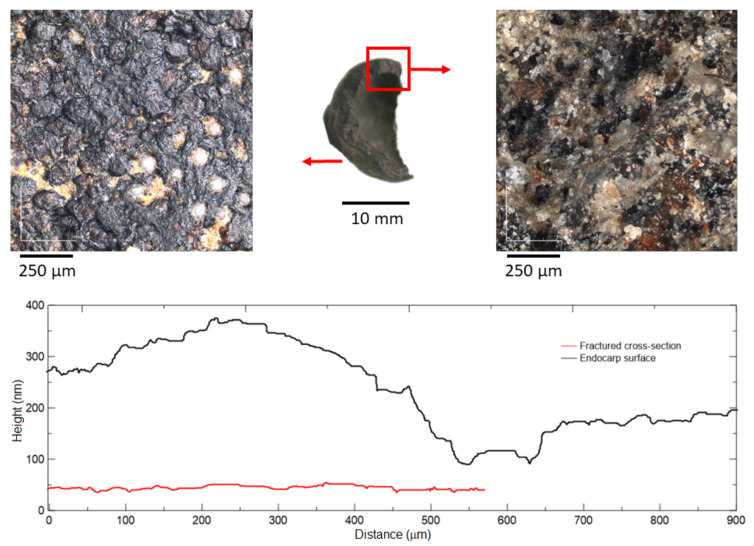
Laser microscopy of endocarp’s surface: roughness and topographic profiles.

The presence of Silicon (Si) accumulation areas was observed by light and laser microscopy, as shown in Figure 10, highlighted by orange arrows. Based on the topographic profile of a single sample, the endocarp’s surface is considerably rough, especially when compared to its fractured cross-section; the average roughness, R${}_{a}$, of the endocarp’s fractured cross-section and surface is, respectively, 24 and 313 μm. When compared to mineral aggregates, the bio-aggregate’s surface presents the highest roughness, as granite, basalt, quartzite and gneiss present R${}_{a}$ values of 202, 175, 145, 95 μm, respectively [47]. Even presenting a high R${}_{a}$ value on its surface, the endocarp will eventually present at least one side with a smooth surface (fractured areas), which may decrease its bonding with the cementitious matrix.

#### 4.2.2. MicroCT scan

**Figure 11:**
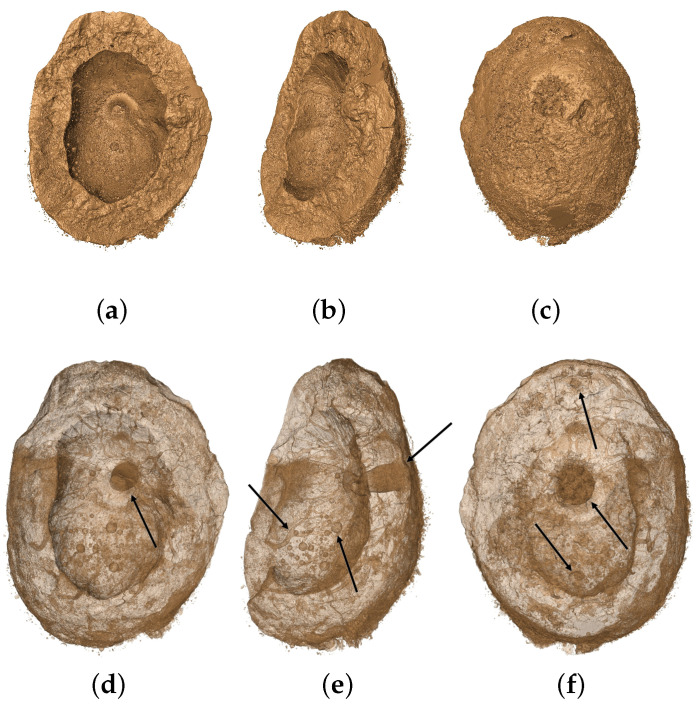
Crushed endocarp’s microCT scan images: opaque-colored/translucent-colored volumes’ (**a**,**d**) inner views, (**b**,**e**) lateral views and (**c**,**f**) outer views.

MicroCT scans are in agreement with light and laser microscopy analysis, as the image results indicate a complex shape and surface of the macauba’s endocarp (A single sample was used for each analysis). With a rotating view of the bio-aggregate, Figure 11a–c present an opaque-colored volume of the inner and outer surfaces, while Figure 11d–f show the translucent-colored version of the same volume, which allow one to observe the endocarp’s internal structure, with its germination pores and their respective canals [48] highlighted by black arrows.

**Figure 12:**
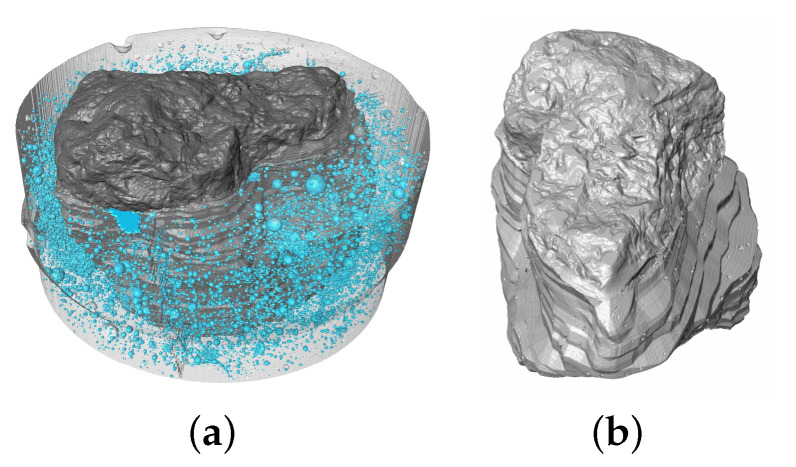
Crushed granite’s microCT scan images: (**a**) embedded in cement paste cylinder, and (**b**) views of the aggregate’s exposed surface.

Figure 12a shows the mineral coarse aggregate embedded in a cement paste, while Figure 12b shows a rotated view of the irregular surface of the rock. Since both aggregates’ images have the same resolution, it is possible to assume that the mineral aggregate have a smoother surface.

**Figure 13:**
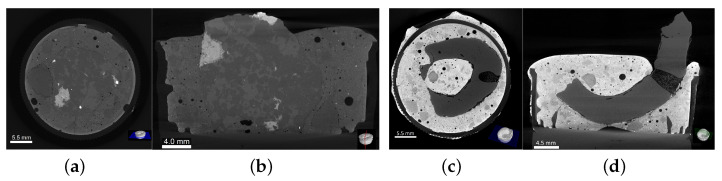
Aggregates embedded in cement paste cylinder: crushed granite’s (**a**) cross- and (**b**) longitudinal sections, and crushed endocarp’s (**c**) cross- and (**d**) longitudinal sections.

Two-dimensional slices of both aggregates embedded in cement paste cylinders are shown in Figure 13 with their respective cross- and longitudinal sections. The mineral aggregate’s slices (Figure 13a,b) are shown under a homogeneous gray scale due to similar density between materials; the absence of voids along the perimeter of the matrix-aggregate interface indicates an effective chemical interaction, and, consequently, an effective adhesion. As the bio-aggregate and the cement paste present different densities, a clear contrast between materials can be observed in Figure 13c,d. It is noteworthy to mention the bio-aggregate’s mechanical interlock and friction due to its semi ellipsoidal shape and surface roughness, as well as voids in the matrix, and localized detachment along its perimeter. The last effect is most probably due to the shrinkage of endocarp.

### 4.3. Mechanical Analyses

When developing a regular concrete dosage, the aggregate strength is rarely tested, because it generally does not influence the overall strength [49] as much as the paste-aggregate bond. Therefore, the following analyses characterize the mechanical behavior of the aggregate-matrix interactions.

#### 4.3.1. Compressive Strength Test of the Cement Paste

The results from the five cast specimens indicate that the cement paste presented a compressive strength of 20.1 ± 1.3 MPa at the age of 7 days, with a Young Modulus of 25.7 ± 3.4 GPa.

#### 4.3.2. Compressive Strength Test of the Bio-Aggregate

**Figure 14:**
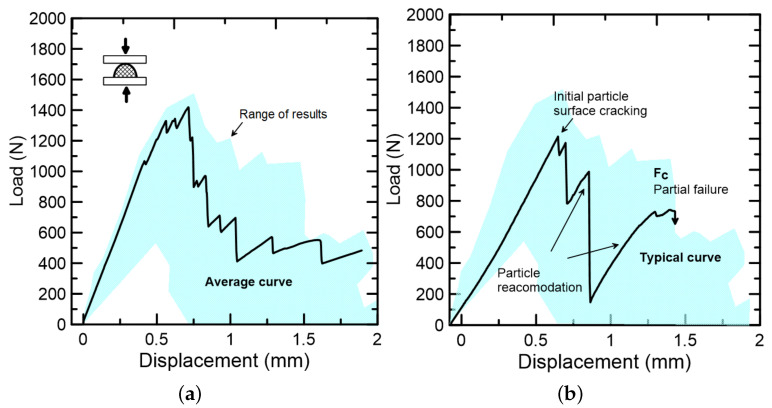
Crushed endocarp submitted to compressive test: (**a**) average curve and range of results, and (**b**) typical curve with mechanical behavior description up to failure.

Figure 14a shows the average curve of the endocarp under compressive loading with its range of results, while Figure 14b shows the endocarp’s mechanical behavior during the compressive strength test: firstly, a linear ascending branch, with an initial particle cracking at peak load, followed by some particle accommodations as displacement continues, up to the failure. All 32 tested specimens presented brittle failure (see test setup in Figure 5). The wide range of results, as well as the observed particle accommodations, were credited to three main reasons: (I) external, from the test setup; (II) geometric, from the irregular semi ellipsoidal shape; and (III) internal, from to the pores and canals, described in Section 4.2.2. The average compressive strength was 3.01 ± 1.63 MPa (for an average maximum compressive load of 1.49 ± 0.81 kN).

#### 4.3.3. Pull-Out Test of the Aggregates

**Figure 15:**
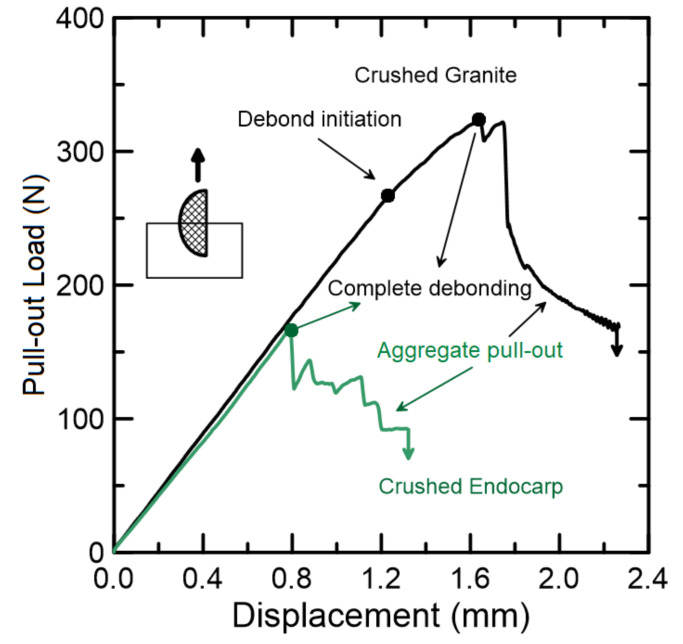
Typical pull-out curves for crushed granite and endocarp.

The pull-out behavior of both aggregates is presented in Figure 15; each type of aggregate comprised 13 specimens. The crushed granite presented a typical pull-out curve with two distinct stages: (I) debond initiation at approximately 270 N, which is indicated by the slope decrease, and (II) complete debonding at peak load—approximately 320 N, which is followed by an abrupt descending branch ruled by frictional forces. Although smaller in magnitude, the crushed endocarp presented a linear ascending branch with the same slope as the crushed granite; with coincident value for debonding and peak load, a smoother descending branch, ruled not only by frictional forced but also by mechanical anchorage, was observed. As expected, the adhesion of the mineral aggregate was higher when compared to the bio-aggregates, with the endocarp showing 55% of granite’s pull-out load. However, due to the endocarp’s shape and surface, the post-peak behavior presented a gradual decrease.

The stiffness at the linear phase is governed by the chemical interaction between cement hydrates and endocarp’s surface. Since mucilage, waxes and saccharides still remain on the endocarp’s surface, the hydration degree, and hence adhesion, is lower in comparison to the crushed granite. However, a smoother post-peak behavior was observed for the bio-aggregate, thanks to the endocarp’s mechanical anchorage and friction, caused by its shape and surface’s irregularities, respectively. With this magnitude of bonding decrease, as well as the bio-aggregate’s compressive strength, it is expected to affect the compressive strength of the cementitious composite. Further investigation to improve the bonding behavior is ongoing, and so far this type of concrete is to be expected to be applied in non-structural purposes, such as precast paving elements, such as pavers and curbs.

### 4.4. Numerical Modeling

Amongst the available interface models, the Coulomb Friction best represented the mechanics of the pull-out test. With cohesion and tensile strength with the same value, as well as friction and dilatancy angles attributed to null, pure shear behavior was guaranteed.

**Figure 16:**
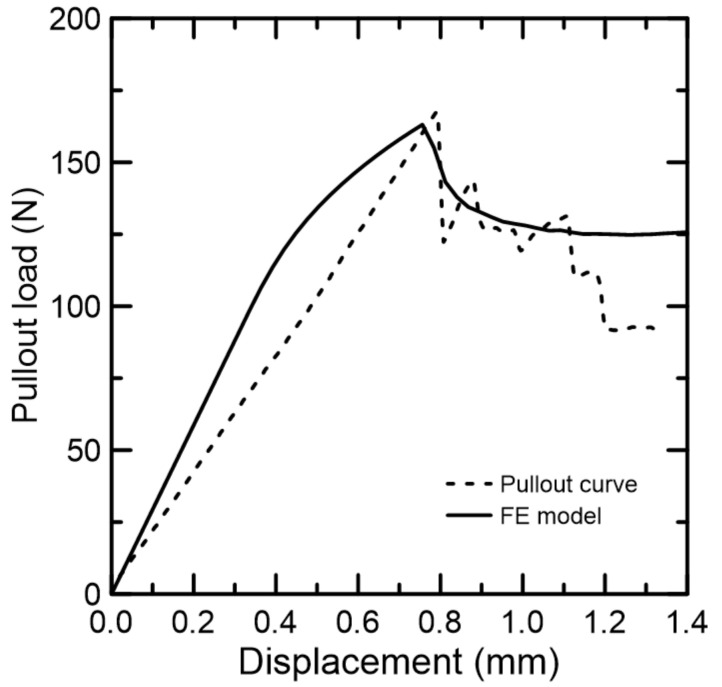
Simulated and experimental pull-out curves.

The pull-out curve of the model is shown in Figure 16. In comparison to the experimental curve (dashed lines), the finite element model was able to satisfactorily simulate the pull-out behavior up to the displacement of 1.1 mm, when it diverged. From this model, it is expected to extract important parameters, such as interface’s tensile strength and cohesion magnitudes, to be used in life-size structures with this type of eco-friendly material.

Although the simulation provided a fair evidence of the experimental behavior, the following limitations are worth listing: the endocarp’s surface and shape irregularities were simplified to a semi ellipsoidal-shaped shell with a smooth surface with the bond-slip mechanisms relying on the interface model parameters only; the ascending curve, although it reaches peak load with approximately 3.5% of error, is unable to behave linearly as the experiment does; the post-peak descending curve tends to a constant pull-out force with the increase in displacement.

## 5. Conclusions

An experimental program and a numerical approach for the macaúba endocarp as a bio-aggregate was presented. It was demonstrated to be a thriving material to be further explored and applied as eco-friendly construction materials. Thus, the following statements can be drawn:

- The high amount of lignin (39%) and hemicelluloses (52%) in the endocarp can retard cement hydration, a chemical process that can be mitigated by accelerators, such as calcium hydroxide;
- The endocarp water absorption of 9% was considered lower when compared to other bio-aggregates and wood (150–200%), which led to lower shrinkage. This lower dimensional variation is an indicative of good adhesion between cement and endocarp;
- Although the crushed endocarp presented lower bonding to the cement paste when compared to the crushed granite (55% of its peak load), its surface showed fair compatibility with the cement paste, with a wider softening curve, due to its hemispherical shape;
- Up to the endocarp complete debonding at approximately 175 N, a similar linear elastic branch between the endocarp and the granite was observed. This behavior was found to be an interesting outcome, not only for being an indication of bonding quality, but also to show that not all conventional lignocelulosic materials present a premature debonding to the cement matrix, as long as a lower shrinkage property is presented;
- The finite element model was able to satisfactorily simulate the pull-out behavior up to 80% of the experiment, when it started to deviate from the experimental results. It was possible to determine parameters that were not possible to directly measure along the experiments, i.e., the magnitudes of interface’s tensile strength and cohesion.

## Figures and Tables

**Table 1:** Overview procedures used to analyze and evaluate the cement-based matrix-aggregate interfaces.

			Aggregate	
**Group**	**Test**	**Standards and/or References**	**Type**	**Modification**
2	TT, ST	[16,17,18,19]	Natural limestone	Prismatic-shaped
2	TT, DST	[15]	limestone aggregates	Prismatic-shaped
2	PPT, BT	[20,21]	Limestone and granite	-
1–3	PSD, SEM-EDS, XRD, CT	[22]	Basalt and limestone	-
1	SEM, X-ray CT analysis	[23,24]	-	-
1,2	LSM, Nano SEM, MBT	[25,26]	Limestone and quartzite	Polishment and shattering

Note: TT = Tensile Test, ST = Shear Test, DST = Direct Shear Test, PPT = Pull-a-Part Test, BT = Brazilian Test, PSD = Pore Size Distribution, SEM = Scanning Microscope, XRD = X-ray Diffraction, CT = Compressive Test, LSM = Microscope, Modified Bending Test.

**Table 2:** Chemical composition of type CPV-ARI cement.

Chemical and Physical Properties	Value
CaO (%)	63.8
SiO${}_{2}$ (%)	17.8
SO${}_{3}$ (%)	3.82
Al${}_{2}$O${}_{3}$ (%)	4.98
K${}_{2}$O (%)	0.82
TiO${}_{2}$ (%)	0.21
MnO (%)	0.10
Others (%)	8.47
Density (g/cm${}^{3}$)	3.2
Superficial Area (m${}^{2}$/kg)	420

**Table 3:** Cement paste properties.

Parameter	Value
Mean Young’s Modulus, $E_{cm}$ (MPa)	25,700
Poisson’s ratio, ν	0.2
Mean tensile strength, $f_{t}$ (MPa)	2.2
Mode-I tensile fracture energy, $G_{f-I}$ (N/mm)	0.13
Crack bandwidth spectification	rots
Crack model	Total strain
Crack orientation	Rotating
Tensile curve	Hordijk
Compression curve	fib 2010 Model Code
Mean compressive strength, $f_{c}$ (MPa)	20
Strain at maximum stress	0.0035
Strain at ultimate stress	0.0040

**Table 4:** Interface parameters.

Parameter	Value
Normal stiffness modulus, $K_{N,z}$ (N/mm${}^{3}$)	0.375
Shear stiffness modulus, $K_{t,x}$ (N/mm${}^{3}$)	0.500
Shear stiffness modulus, $K_{t,y}$ (N/mm${}^{3}$)	0.500
Material model	Coulomb friction
Cohesion, c (MPa)	0.17
Friction angle, ϕ (degrees)	1 $\times \;10^{-5}$
Dilatancy angle, ψ (degrees)	0.00
Interface opening model	Gapping model
Tensile strength, $f_{t}$ (MPa)	0.17
Mode-II shear	Brittle

**Table 5:** Dimensions of the aggregates.

	Crushed Granite	Crushed Endocarp
	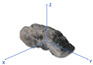	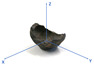
Thickness (Z, mm)	6.3	0.6
Length (Y, mm)	16.2	20.5
Width (X, mm)	9.3	20.2
L/T	2.5	34.2
W/T	1.5	12.1

**Table 6:** Physical properties of the crushed endocarp.

Water Absortion (%)			
Raw (Coarse)	Shrinkage (%)	Density (g/cm³)	**pH**
9.05 ± 0.12	6.75 ± 1.14	1.23 ± 0.1	5.24

**Table 7:** Chemical composition of the crushed endocarp.

Total Extractives (%)	Insoluble Lignin (%)	Holocellulose (%)
3.10 ± 0.26	39.60 ± 0.16	52.64 ± 0.10

## Data Availability

The authors declare that all data supporting the findings of this study are available within the article. Additional information are available from the corresponding author upon request.
